# Editorial for the Special Issue on Micromachines for Dielectrophoresis, Volume II

**DOI:** 10.3390/mi14040769

**Published:** 2023-03-30

**Authors:** Rodrigo Martinez-Duarte

**Affiliations:** Multiscale Manufacturing Laboratory, Department of Mechanical Engineering, Clemson University, Clemson, SC 29634, USA; rodrigm@g.clemson.edu

Dielectrophoresis (DEP) remains an effective technique for the label-free identification and manipulation of targeted particles ranging from sizes from nano to micrometers and from inert particles to biomolecules and cells. This Special Issue includes 13 novel contributions to the field in aspects ranging from fundamentals and theoretical modeling to applications.

Several contributions advance the DEP fundamentals in a significant manner. In Protein Dielectrophoresis: A Tale of Two Clausius–Mossottis—Or Something Else? [1], Ron Pethig postulates an empirical theory to predict the DEP response of a protein from the magnitude of the dielectric *β*-dispersion produced by its relaxing permanent dipole moment using the equivalent of a molecular version of the macroscopic Clausius–Mossotti factor. His contribution responds to the observation that the standard DEP theory fails to describe the DEP data obtained for different proteins in the past several years. In the first of two related contributions, Gimsa in Active, Reactive, and Apparent Power in Dielectrophoresis: Force Corrections from the Capacitive Charging Work on Suspensions Described by Maxwell–Wagner’s Mixing Equation [2], introduces a new expression for the DEP force derived from the electrical work in a charge-cycle model that allows the field-free transition of a single object between the centers of two adjacent cubic volumes in an inhomogeneous field. The comparison of this expression with the classical solution provides a new perspective on the notorious problem of electrostatic modeling of AC electrokinetic effects in lossy media and provides insight into the relationships between active, reactive, and apparent power in DEP force generation. In a second contribution, Gimsa and Radai in Dielectrophoresis from the System’s Point of View: A Tale of Inhomogeneous Object Polarization, Mirror Charges, High Repelling and Snap-to-Surface Forces and Complex Trajectories Featuring Bifurcation Points and Watersheds [3] build upon the first contribution to describe the polarizability of an entire DEP system as a function of the position of the object with a numerical “conductance field” and argue that such an approach can explain experimental findings, such as the accumulation of viruses and proteins, where the dipole approach cannot account for sufficiently high holding forces to defeat the Brownian motion. Another important contribution is that from Zaman et al. titled Modeling Brownian Microparticle Trajectories in Lab-on-a-Chip Devices with Time Varying Dielectrophoretic or Optical Forces [4], where they present a generalized computational physics model to simulate the trajectories of particles under the influence of both DEP and optical forces. Of note, their model considers time varying applied forces, Brownian motion, fluid flow, collision mechanics, and hindered diffusion caused by hydrodynamic interactions and is shown to be capable of simulating the time evolution of the positions and velocities of an arbitrarily large number of particles simultaneously. For the benefit of the community, this model is made freely available through a GitHub repository. Miloh and Nagler present Travelling-Wave Dipolophoresis: Levitation and Electrorotation of Janus Nanoparticles, which is a theoretical study of the hydrodynamic and electrokinetic response of both metallic spherical polarized colloids as well as metallodielectic Janus particles. In this work, they consider cases of linear and helical time-harmonic travelling-wave excitations and provide explicit expressions for the resulting DEP and charge-induced [5] electrophoretic forces and moments exerted on freely suspended particles. Last, Williams et al. present their work on the observation of simultaneous DEP and electrostatic forces in the implementation of an electric curtain. In Particle-Induced Electrostatic Repulsion within an Electric Curtain Operating below the Paschen Limit [6], they describe how an electric curtain is implemented and polarized to produce an electric field below the Paschen limit. Experiments conducted with micrometer-sized soda lime glass beads show how individual particles themselves can trigger electrostatic repulsion in an otherwise dielectric system.

Important advances on different applications are also reported. In Dielectrophoretic Micro-Organization of Chondrocytes to Regenerate Mechanically Anisotropic Cartilaginous Tissue [7], Takeuchi and Miyata leverage negative DEP to pattern Bovine chondrocytes into line-array cell clusters in an agarose gel. Their results show how the embedded chondrocytes remained viable and reconstructed cartilaginous tissue along the patterned cell array and how the cell-containing agarose gel demonstrated mechanical anisotropy. In One-Dimensional Flow of Bacteria on an Electrode Rail by Dielectrophoresis: Toward Single-Cell-Based Analysis [8], Yamaguchi and Yamamoto describe the numerical and experimental evaluation of a device utilizing a DEP force to array bacteria in a single line to allow their facile numeration, a feat desired in biotechnology and medicine to count bacteria with single-cell resolution. In A Study of Dielectrophoresis-Based Liquid Metal Droplet Control Microfluidic Device [9] by Tian et al., an array of liquid gallium-based alloy electrodes driven at ±1000 V is demonstrated to control a liquid metal droplet, physically isolated from the electrodes, at velocities up to 1 mm/s and depending on the droplet diameter. Towards the use of DEP in practical applications, Ettehad and Wenger in Characterization and Separation of Live and Dead Yeast Cells Using CMOS-Based DEP Microfluidics [10] present a DEP system enabled by CMOS to separate viable from non-viable cells. This is significant given the wide application of CMOS fabrication processes in everyday electronics. Moving into the manipulation of nanometric objects using DEP, Dimaki and co-authors in Sub–100 nm Nanoparticle Upconcentration in Flow by Dielectrophoretic Forces [11] present a novel microfluidics system for the concentration of sub–100 nm nanoparticles in a flow using electrical forces generated by a DC or AC electrical field. The authors show how using different electrode configurations can lead to the concentration of particles, as low as 47 nm, by a factor of up to 11. In Dielectrophoresis-Based Positioning of Carbon Nanotubes for Wafer-Scale Fabrication of Carbon Nanotube Devices [12], Kimbrough et al. report the use of DEP for the wafer-scale fabrication of carbon nanotube field-effect transistors, or FET. They note a relatively high rate of FET functionality, up to 87%, in a DEP-based process that is amenable to integration with processes used in semiconductor manufacturing. Last, in Design of Driving Waveform Based on a Damping Oscillation for Optimizing Red Saturation in Three-Color Electrophoretic Displays [13], Yi et al. present a study on the waveform to drive three-color electrophoretic displays (EPDs), particularly their red saturation. To this end, they present an optimized waveform composed of multiple stages, including erasing, particle activation, the purification of red particles, and the display of red color. The experimental results show how the maximum red saturation reaches up to 27.57% improvement compared with more traditional driving waveforms.

I encourage the reader to explore these excellent and meaningful contributions to the field. I also wish to express thanks to all authors for contributing to this second installment of /Micromachines/ for Dielectrophoresis; the reviewers, whose insightful feedback helped improve the impact of these contributions; and the Academic Editors Antonio Ramos, Xiangchun Xuan, Nam-Trung Nguyen, and Aiqun Liu for their important feedback.
